# Compressed sensing MRI: a review from signal processing perspective

**DOI:** 10.1186/s42490-019-0006-z

**Published:** 2019-03-29

**Authors:** Jong Chul Ye

**Affiliations:** Department of Bio and Brain Engineering, Korea Adv. Inst. of Science & Technology (KAIST), 291 Daehak-ro, Daejeon, Korea

**Keywords:** MRI; compressed sensing; k-space

## Abstract

Magnetic resonance imaging (MRI) is an inherently slow imaging modality, since it acquires multi-dimensional k-space data through 1-D free induction decay or echo signals. This often limits the use of MRI, especially for high resolution or dynamic imaging. Accordingly, many investigators has developed various acceleration techniques to allow fast MR imaging. For the last two decades, one of the most important breakthroughs in this direction is the introduction of compressed sensing (CS) that allows accurate reconstruction from sparsely sampled k-space data. The recent FDA approval of compressed sensing products for clinical scans clearly reflect the maturity of this technology. Therefore, this paper reviews the basic idea of CS and how this technology have been evolved for various MR imaging problems.

## Background

Magnetic resonance imaging (MRI) exploits the nuclear magnetic resonance (NMR) phenomena to enable high contrast imaging. Since the first observation of NMR absorption in a molecular beam in 1938, the main research thrust was to understand and utilize the NMR phenomena for spectroscopy applications. Then, Lauterbur introduced in 1973 the gradient field to encode the spatial origin of the radio waves emitted from the nuclei of the object. This breakthrough allowed for multi- dimensional imaging by NMR physics.

The idea of Lauterbur can be easily understood using k-space interpretation that describes MR sampling as Fourier encoding in 2D or 3D spaces. Specifically, data collected by an MRI scanner are samples of the spatial Fourier transform of an object image. Hence, in order to obtain an image without aliasing artifacts, k-space samples need to satisfy the Nyquist sampling criterion.

Despite this close link to signal sampling theory, until the first demonstration of the sensitivity encoding (SENSE) technique by Prussemann [1], MR imaging was not considered as an important research topic for signal processing. Specifically, Prussemann et al. [1] showed that spatial diversity information from coil sensitivity maps have additional information that can be exploited for fast signal acquisition. Furthermore, Sodickson et al. [2] proposed the simultaneous acquisition of spatial harmonics (SMASH). These works gave a birth of parallel imaging and iterative reconstruction methods, and has resulted in a flurry of novel ideas and algorithms, including Generalized Autocalibrating Partially Parallel Acquisitions (GRAPPA) by Griswold [3] and k-t space method for cardiac imaging such as [4–9].

The common theme in these approaches is that the data redundancy can be exploited to reduce the required sampling rate. Because redundant data can be compactly represented in some transform domains, it is also closely related to the concept of “sparsity”. Originally investigated by Bresler and his students for on- the-fly Fourier imaging [10, 11], the sparsity regularization has become the main workhorse in modern accelerated MRI researches thanks to the introduction of the compressed sensing theory [12, 13]. Ever since the first demonstration of compressed sensing MRI by Lustig et al. [14, 15], the compressed sensing MRI has become the essential tools in modern MR imaging researches. In this paper, we will review these ideas in more detail.

## Main text

### MR forward models

Before we introduce the concept of compressed sensing MR, we begin with the discussion of MR forward model. Mathematically, the forward model for k-space measurement can be described by.

1 $$ {b}^i\left(\mathbf{k}\right)=\int {\gamma}^i(r){e}^{-j2\pi {k}^Tr} dr,\kern1em i=1,\dots, C, $$

where **r** ∈ R^d and **k** ∈ R^*d*^, d = 2, 3 denote the image domain and k-space coordinates, respectively, C is the number of the coil and the i-th coil image γ^*i*^ is given by

2 $$ {\upgamma}^i\left(\mathbf{r}\right)=\upnu \left(\mathbf{r}\right){s}^i\left(\mathbf{r}\right) $$

Here, ν(**r**) denotes the contrast weighted bulk magnetization distribution, and *s*^*i*^(**r**) is the corresponding coil sensitivity of the i-th coil. Although the expression may give an impression that the measurement is two- or three- dimensional, this is indeed originated from a 1-D measurement since the k-space trajectory is a function of time, i.e. **k**:= **k**(*t*), and we acquire one k-space sample at each time point t. This makes the MR imaging inherently slow, since we need to scan through a 3-D object via 1-D trajectories.

Dynamic MRI is another important MR technique to monitor dynamic processes such as brain hemodynamics and cardiac motion. Among the various forms of dy- namic MR modeling, here we mainly focus on the k-t formulation. Specifically, consider a discrete imaging equation for cartesian trajectory for simplicity. Because the samples along the readout direction are fully sampled, most of the dynamic MR formulation is applied separably after taking the Fourier transform along the read- out direction. More specifically, let γ*(s, t)* denote the unknown image content (for example, proton density, T1/T2 weighted image, etc.) on the spatial coordinate s along the phase encoding line at time instance t. Then, the k-t space measurement *b(k, t)* at time t is given by

3 $$ b\left(k,t\right)=\int \gamma \left(s,t\right){e}^{-j2\pi ks} ds $$

where γ*(s, t)* is the spatio-temporal image which may be weighted by coil sensitivity map for the case of parallel imaging.

Throughout the paper, for simplicity we often use the operator notation for (1):

$$ {b}^i=F\left[{\mathrm{S}}^i\right]\upnu, \kern1em i=1,\cdots, \mathrm{C}, $$

where [S^i^] is a diagonal operator comprised of the i-th coil sensitivity. Similarly, we use operator notation for (3).

4 $$ b=F\upgamma $$

or

5 $$ {b}^i=F{\gamma}^i,\kern1em \mathrm{i}=1,\cdots, \mathrm{C} $$

for the case of parallel imaging.

### Compressed sensing theory

#### Performance guarantees

Compressed sensing (CS) theory [12, 16, 17] addresses the accurate recovery of unknown sparse signals from underdetermined linear measurements and has become one of the main research topics in the signal processing area for the last two decades [18–23]. Most of the compressed sensing theories have been developed to address the so-called single measurement vector (SMV) problems [12, 16, 17]. More specifically, let m and n be positive integers such that m < n. Then, the SMV compressive sensing problem is given by

6 $$ (P0):{\displaystyle \begin{array}{c}\operatorname{minimize}\ {\left\Vert \mathrm{x}\right\Vert}_0\\ {}\mathrm{subject}\ \mathrm{to}\ \left\Vert \mathrm{y}-\mathrm{Ax}\right\Vert <\upepsilon, \end{array}} $$

where y ∈ R^m, A ∈ R^*m*\×*n*, x ∈ R^n, and ϵ denotes the noise level. (P0) implies that the solution favours the sparsest solution.

Since (P0) requires a computationally expensive combinatorial optimization, greedy methods [24], reweighted norm algorithms [25, 26], or convex relaxation using the l_1_ norm [12, 27] have been widely investigated as alternatives. In particular, the convex relaxation approach addresses the following l_1_ minimization problem:

7 $$ (P1):{\displaystyle \begin{array}{c}\operatorname{minimize}\ {\left\Vert \mathrm{x}\right\Vert}_1\\ {}\mathrm{subject}\ \mathrm{to}\ \left\Vert \mathrm{y}-\mathrm{Ax}\right\Vert <\upepsilon, \end{array}} $$

One of the important theoretical tools of CS is the so-called restricted isometry property (RIP), which enables us to guarantee the robust recovery of certain input signals [17]. More specifically, a sensing matrix $\mathrm{A}\in {\mathrm{R}}^{m\times \mathrm{n}}$ is said to have a k- restricted isometry property (RIP) if there is a constant 0 ≤ δ_*k*_ < 1 such that

$$ \left(1-{\updelta}_k\right)\left\Vert \mathrm{x}\right\Vert 2\le \left\Vert \mathrm{Ax}\right\Vert 2\le \left(1+{\updelta}_k\right)\left\Vert \mathrm{x}\right\Vert 2. $$

for all x ∈ R^n with ||x||_0_ ≤ k. It has been demonstrated that δ_2*k*_ < $\sqrt{2}-1$ is sufficient for l_1_/l_0_ equivalence [12]. For many classes of random matrices, the RIP condition is satisfied with high probability if the sampling pattern is incoherent and the number of measurements satisfies m ≥ *ck* log(*n/k*) for some constant c > 0 [17].

#### Optimization approaches

In practice, the following unconstrained form of optimization problem is often used:

8 $$ \underset{x}{\min}\frac{1}{2}{\left\Vert \mathrm{y}-\mathrm{Ax}\right\Vert}^2+\uplambda {\left\Vert \Psi \mathrm{x}\right\Vert}_1 $$

where y is noisy measurement, λ is the regularization parameter, and Ψ refers to an analysis transform such that Ψx becomes sparse. Note that for the special case of Ψ = I, (8) is reduced to (*P*1).

One of the technical issues in solving (8) is that the cost function is not smooth at Ψx = 0, which makes the corresponding gradient ill-defined. This leads to the development of various techniques, which are culminated as the new type of convex optimizaton theory called *proximal optimization* [28]. For example, the popular op- timization methods such as forward-backward splitting (FBS) [29], split Bregman iteration [30], alternating directional method of multiplier (ADMM) [31], Douglas- Rachford splitting algorithm [32], and primal-dual algorithm [33] have been devel- oped to solve compressed sensing problems. However, the comprehensive coverages of these techniques deserves another review paper, so in this section we mainly review the ADMM algorithms which have been extensively used for compressed sensing MRI ever since its first introduction to compressed sensing MRI [34].

More specifically, we convert the problem (8) to the following constraint problem:

9 $$ \underset{\gamma, \mathrm{u}}{\min }\ \frac{1}{2}{\left\Vert y- A\gamma \right\Vert}^2+\uplambda {\left\Vert u\right\Vert}_1 $$

subject to

10 $$ \mathrm{u}=\Psi \upgamma . $$

Then, the associated ADMM is given by

$$ {\gamma}^{\left(k+1\right)}=\mathit{\arg}\ \underset{x}{\min }\ \frac{1}{2}{\left\Vert y- A\gamma \right\Vert}^2+\frac{\upmu}{2}{\left\Vert {\upzeta}^{(k)}+\Psi \upgamma -{\mathrm{u}}^{(k)}\right\Vert}^2 $$

$$ {u}^{\left(k+1\right)}=\mathit{\arg}\ \underset{u}{\min}\kern0.50em \uplambda {\left\Vert u\right\Vert}_1+\frac{\upmu}{2}{\left\Vert {\upzeta}^{(k)}+\Psi {\upgamma}^{\left(k+1\right)}-\mathrm{u}\right\Vert}^2 $$

$$ {\upzeta}^{\left(k+1\right)}={\upzeta}^{(k)}+{x}^{\left(k+1\right)}-{u}^{\left(k+1\right)}, $$

Now, each step of ADMM has a closed-form solution. Algorithm 1 summarises the resulting ADMM iteration, where shrink_1_ denotes the soft-tresholding:

11 $$ {shrink}_1\left(\mathrm{x},\uptau \right)=\operatorname{sgn}\left(\mathrm{x}\right)\ \max \left\{0,|\mathrm{x}|-\uptau\ \right\}, $$

for the threshold value τ > 0.

In addition to the analysis prior in (8), the total variation (TV) penalty has been also extensively used for imaging applications, because the finite difference operator

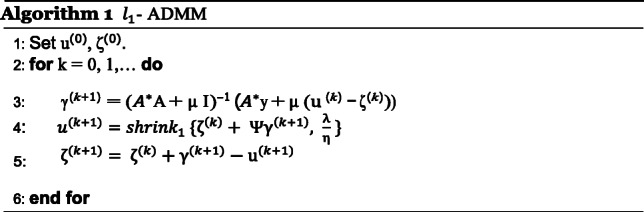



can sparsify smooth images. Specifically, the TV minimisation problem assumes the following form:

12 $$ \underset{x}{\min}\frac{1}{2}{\left\Vert y- Ax\right\Vert}^2+\uplambda \mathrm{TV}\left(\mathrm{x}\right), $$

where T V (x) is given by

13 $$ \mathrm{TV}\left(\mathrm{x}\right)={\left\Vert \nabla x\right\Vert}_{1,p}, $$

where ‖∙‖_1, *p*_, *p* = 1, 2 deotes the l_1_/l_*p*_-mixed norm. In d-dimensional space (e.g. d = 2 for images), the discretized implementation can be defined as

$$ {\left\Vert \nabla x\right\Vert}_{1,p}={\sum}_{i=1}^n{\left\Vert \nabla x(i)\right\Vert}_p $$

where ∇x(i) ∈ R^*d*^ denotes the gradient of x at the i-th coordinate and n denotes the number of discretizated samples.

In order to apply ADMM for (12), we need to focus on the primal formulation of the total variation penalty:

14 $$ TV(x)={\left\Vert \nabla x\right\Vert}_{1,p}={\sum}_{i=1}^n{\left\Vert \nabla x(i)\right\Vert}_p $$

Now, we define a splitting variable u(i) = ∇x(i) ∈ R^*d*^. Then, the constraint opti- mization formulation is given by

15 $$ \underset{x,{\left\{u(i)\right\}}_{i=1}^n}{\min}\frac{1}{2}{\left\Vert y- Ax\right\Vert}^2+\uplambda {\sum}_{i=1}^n{\left\Vert u(i)\right\Vert}_p $$

16 $$ \mathrm{Subject}\ \mathrm{to}\ \mathrm{u}\left(\mathrm{i}\right)=\nabla x(i),i=1,\cdots, n $$

Then, the associated ADMM is given by

$$ {x}^{\left(k+1\right)}=\mathit{\arg}\ \underset{x}{\min }\ \frac{1}{2}{\left|\left|y- Ax\right|\right|}^2+\frac{\upeta}{2}{\sum}_i{\left\Vert {\upzeta}^{(k)}(i)+\nabla x(i)-{\mathrm{u}}^{(k)}(i)\ \right\Vert}^2 $$

$$ {u}^{\left(k+1\right)}(i)=\mathit{\arg}\ \underset{u(i)}{\min}\kern0.50em \uplambda {\left\Vert u(i)\right\Vert}_p+\frac{\upeta}{2}{\left\Vert {\upzeta}^{(k)}(i)+\nabla {x}^{\left(k+1\right)}(i)-\mathrm{u}\left(\mathrm{i}\right)\ \right\Vert}^2 $$

$$ {\upzeta}^{\left(k+1\right)}(i)={\upzeta}^{(k)}(i)+\nabla {x}^{\left(k+1\right)}(i)-{u}^{\left(k+1\right)}\left(\mathrm{i}\right) $$

Each step has the closed form expression. Algorithm 2 summarises the TV-ADMM algorithm, where shrink_*vec,*2_(x) for x ∈ R*d* denotes the vector shrinkage [34]:

17 $$ {shrink}_{vec,2}\left(x,\uptau \right)=\frac{x}{\left|x\right|}\max \left\{0,\left|x\right|-\uptau\ \right\},x\in {\mathrm{R}}^d $$

for the threshold value τ > 0.

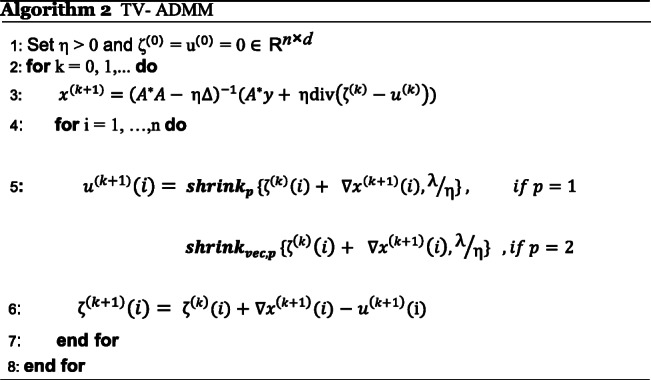



### Basic MR ingredients for compressed sensing

In order to apply compressed sensing theory for specific imaging applications, the unknown signal should be sparse in some transform domain, and the sensing matrix should be sufficiently incoherent. This section shows why these conditions can be readily satisfied in MR imaging, which is one of the main reasons that allows for successful applications of CS theory to MR imaging.

#### Sparsity of MR images

An MR image is rarely sparse in its own. However, one of the important observations that led to the successful applications of CS to MR is that the sparsity is closely related to signal redundancies. This is because redundant signals can be easily converted to sparse signals using some transforms.

**Fig. 1:**
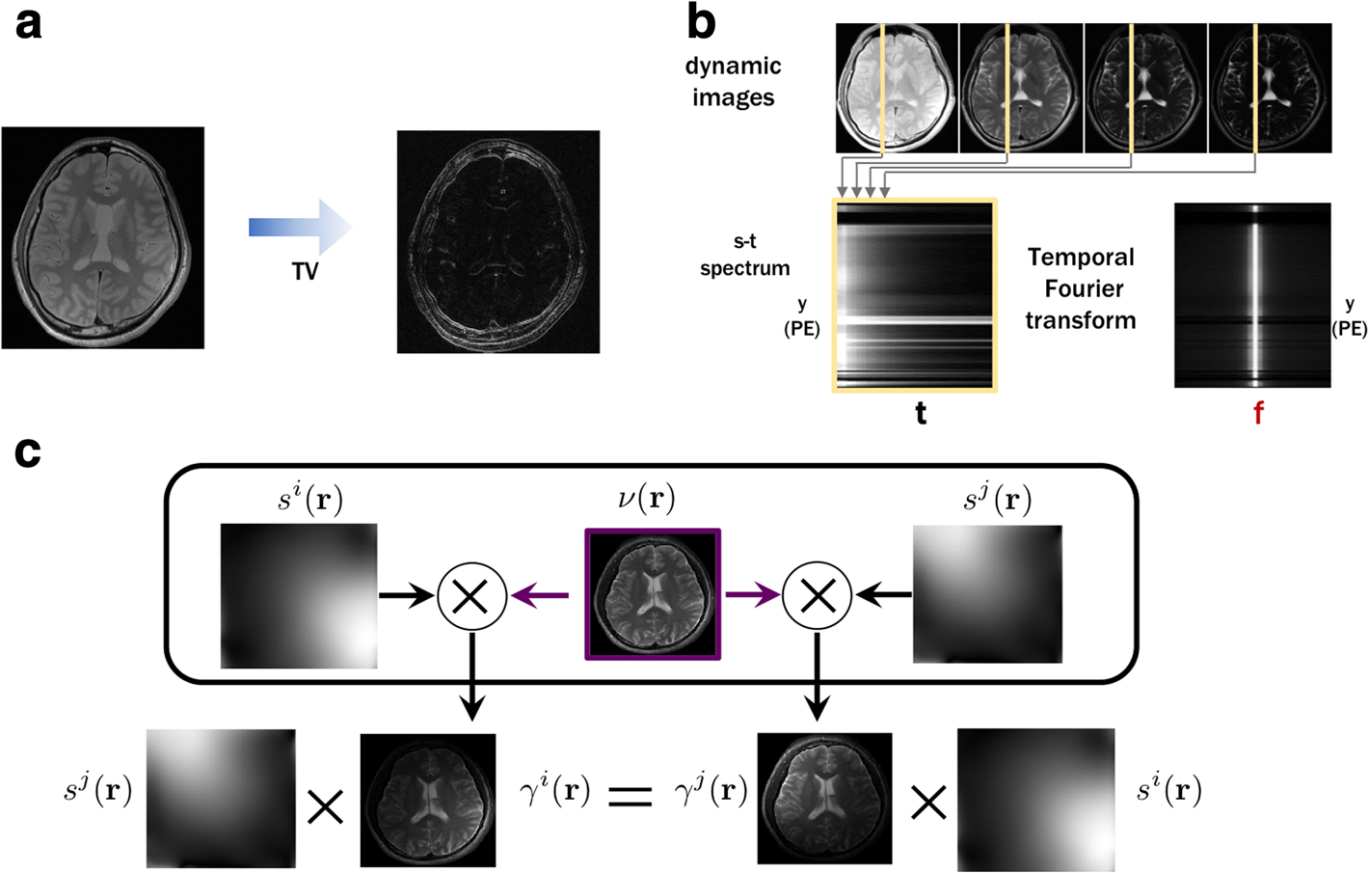
Various types of sparsity in MRI. (**a**) Sparsity from spatial domain redundancy, (**b**) Sparsity from temporal redundancy, and (**c**) sparsity from mu.ti-channel redundancy

Basically, there are three major directions that have been investigated in com- pressed sensing MRI: 1) the spatial domain redundancy, 2) the temporal domain redundancy, and 3) the coil domain redundancy. For example, as shown in Fig. 1(a), natural images can be sparsely represented in finite difference or wavelet transform domain, although the image is not sparse in its own. This observation is the main idea that allows for total variation (TV) and wavelet transform approaches for image denoising, and reconstruction. Accordingly, TV and wavelets have been the main transforms that have been extensively used in most of the CS MRI researches. On the other hand, dynamic MR images such as cardiac cine, functional MRI, and MR parameter mapping have significant redundancy along the temporal dimension as shown in Fig. 1(b). For example, if the image is perfectly periodic, then temporal Fourier transform may be the optimal transform to sparsify the signal. However, in many dynamic MR problems, the temporal variations are dependent on the MR physics as well as specific motion of organs, so the analytic transform such as Fourier transform may not be an optimal solution, but more data-driven approaches such as PCA or dictionary learning are better options. Indeed, these observation leads to dictionary learning approaches that will be discussed later.

On the other hand, the coil redundancy is somewhat distinct compared to the spatial- and temporal- domain redundancy. As shown in Fig. 1(c), the redundancy of the coil image stems from the underlying common images, which results in cross- channel redundancies:

18 $$ {s}^j(r){\gamma}^i(r)={s}^i(r){\gamma}^j(r),\mathrm{i}\ne \mathrm{j},\forall \mathrm{r} $$

where *s*^*i*^ denotes the i-th coil sensitivity map and *γ*^*i*^ is the coil image. The rela- tionship in (18) is easy to show since the coil image is given by (2). The main idea of the classical parallel MRI is to exploit this relationship. Specifically, the sensi- tivity encoding (SENSE) [1] exploits the image domain redundancy described in (18), whereas the k-space domain approaches such as GRAPPA [3] exploits its dual relationship in the k-space:

19 $$ {\widehat{s}}^j(r)\ast {\widehat{\gamma}}^i(r)={\widehat{s}}^i(r)\ast {\widehat{\gamma}}^j(r),\mathrm{i}\ne \mathrm{j},\forall \mathrm{r} $$

where ∗ denotes the convolution, and ${\widehat{s}}^j$, ${\widehat{\gamma}}^j$enote the Fourier transform of *s*^*j*^and *γ*^*j*^respectively. Later we will describe how these two expressions of the coil dimensional redundancies have been exploited in compressed sensing MRI.

#### Incoherent sampling pattern

**Fig. 2:**
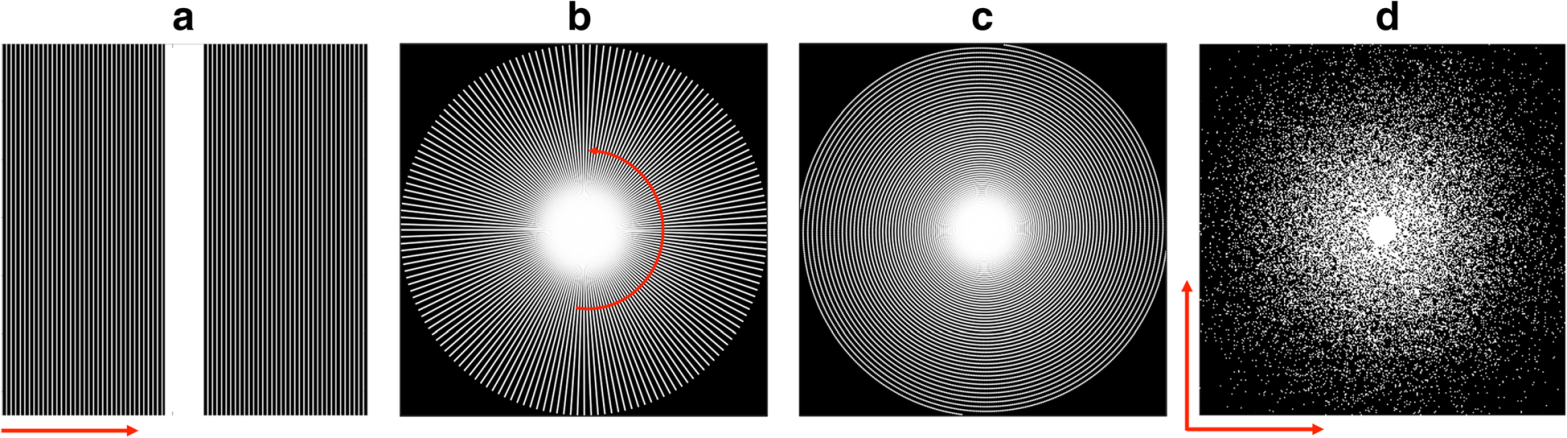
Various under-sampling patterns: (**a**) Cartesian undersampling, (**b**) radial undersampling, and (**c**) spiral undersampling

In compressed sensing MRI, downsampling pattern is very important to impose the incoherence, but its realization is limited by MR physics. For example, in the 2-D acquisition, the readout direction should be fully sampled, so there is only freedom along the phase encoding direction in designing incoherence sampling patterns. Figure 2(a)-(c) shows the examples of realizable sampling patterns that have been exploited in the literature: a) cartesian undersampling, b) radial trajectory, and c) spiral trajectory. In particular, the radial and spiral trajectories, which have been studied even before the advanced of the compressed sensing [35], have more incoherent radial sampling patterns compared to the cartesian undersampling.

On the other hand, if we deal with 3-D imaging or dynamic imaging, there are more rooms for sampling pattern design, since there are two dimensional degree of freedom. Figure 2(d) shows an example of 2-D random sampling pattern. For the case of wavelet transform-based sparsity imposing prior, Lustig et al. [14] showed that the incoherence can be optimized by generalizing the notion of PSF to Transform Point Spread Function (TPSF). Specifically, TPSF measures how a single transform coefficient of the underlying object ends up influencing other transform coefficients of the measured undersampled object. To calculate the TPSF, a single point in the wavelet transform space at the i-th location is transformed to the image space and then to the Fourier space. Once the corresponding Fourier space data is subsampled by the given downsampling pattern, its influence in the wavelet transform domain can be calculated by taking inverse transform followed by the inverse wavelet trans- form. To have the best incoherence property, the side of TPSF should be as small as possible, such that the side lobe contribution can be removed by shrinkage op- eration. This was used as the main criterion for sampling pattern design.

#### Historical milestones

The earliest application of the compressed sensing for MRI was done by Lustig and his collaborators [14]. The application of CS to dynamic MRI was pioneered by Jung et al. [18] and Gamper et al. [36], which was later significantly improved by Jung et al. [20]. The positive results of these earlier works have resulted in flurry of new ideas in static and dynamic MRI.

In terms of combining CS with parallel imaging, the first application for static imaging was done by Block et al. [37] by combining total variation penalty and parallel imaging; and by Liang et al. [38] in more general form using SENSE. In dynamic imaging, the works by Jung et al. [18] and Feng et al. [39] are among the first that combine the SENSE type parallel imaging with the k-t domain compressed sensing. Combination with CS and parallel imaging using GRAPPA type constraints was pioneered by Lustig et al. [40]. Coil compression techniques have been also investigated to reduce the number of coils for CS reconstruction [41]. Feng et al. [42] later combined compressed sensing, parallel imaging, and golden-angle radial sampling for fast and flexible dynamic volumetric MRI, which has been approved for clinical use.

Aside from the standard l_1_ and TV penalty, several innovative sparsity inducing penalty have been used for compressed sensing MRI. For example, Trzasko et al. [43, 44] proposed a direct l_0_ mimimization approaches, whereas Knoll et al. [45, 46] proposed a generalized total variation approaches, and Sung et al. [47] combined the approximate message passing algorithm with parallel imaging. This idea was then extended to the dictionary learning [20, 48, 49] and motion compensation [20, 50]. Then, the idea of low-rank regularization was soon introduced [49, 51–53] thanks to the theoretical advances by Candes et al. [54]. The low-rank idea was further ex- tended to structured low-rank approach for parallel imaging [55] and single coil imaging with the finite support [56]. The duality between the compressed sensing and low-rank Hankel matrix approaches were discovered by Jin and his colleagues [57–60] and Ongie et al. [61]. In particular, the unified framework for compressed sensing and parallel MRI in terms of low-rank Hankel matrix approaches was presented by Jin et al. [57] and its theoretical performance guarantees was also given in [62].

In the following, we provide more detailed reviews of these historical milestones in compressed sensing MRI.

## Basic formulation of compressed sensing MRI

Although some MR images such as angiograms are already sparse in the pixel representation, more complicated images are rarely sparse, but only have a sparse representation in some transform domain, for example, in terms of spatial finite- differences or their wavelet coefficients. Based on this observation, Lustig et al. [14] proposed the first compressed sensing MRI using spatial domain wavelet transform as a sparsifying transform. More specifically, the problem is formulated as

20 $$ \underset{x}{\min }\ {\left\Vert \Psi \upgamma \right\Vert}_1 $$

$$ \mathrm{subject}\ \mathrm{to}\ {\left\Vert y- DF\upgamma \right\Vert}^2<\epsilon $$

where ‖∙‖_1_ denotes the l_1_ norm, *F* is the Fourier transform, γ denotes the 2-D complex image, Ψ is a either finite difference or spatial wavelet transform, *D* is the downsampling pattern, and y is the downsampled k-space measurement. Eq. (20) was solved using a nonlinear conjugate gradient method.

The resulting optimization algorithms are, however, computational expensive. For cartesian sampling trajectory, this problem can be overcome as follows. Specifically, the image update step for (20) in ADMM implementation can be summarized as

$$ \left({F}^{\ast }{D}^{\ast } DF+\mu I\right)\upgamma ={F}^{\ast }{D}^{\ast }y-\mu {\Psi}^{\ast}\left({\upzeta}^{(k)}-{u}^{(k)}\right), $$

which is computationally expensive due to the matrix inverse to obtain γ. Instead of using a direct matrix inverse, by multiplying the Fourier transform *F* to both sides, we have

$$ \left({D}^{\ast }D+\mu I\right)\widehat{\gamma}={D}^{\ast }y+\mu F{\Psi}^{\ast}\left({u}^{(k)}-{\upzeta}^{(k)}\right) $$

where $\widehat{\gamma}=F\upgamma$. Note that a diagonal matrix D*D consisting of ones and zeros. The ones are at those diagonal entries that corresponds to the sampled locations in the k-space. Let Ω denote the sampled location. Then,

21 $$ {\widehat{\gamma}}^{\left(k+1\right)}={P}_{\Omega}\left(\frac{D^{\ast }y+\mu F{\Psi}^{\ast}\left({u}^{(k)}-{\upzeta}^{(k)}\right)}{1+\mu}\right)+{P}_{\Omega^c}\left(F{\Psi}^{\ast}\left({u}^{(k)}-{\upzeta}^{(k)}\right)\right), $$

where P_Ω_ and P_Ω_*c* denotes the projection on the sampling location Ω and its com- plement Ω^*c*^, respectively. Then, the image update *γ*^(*k* + 1)^can be simply done by taking the fast Fourier transform (FFT).

The first experimental demonstration by Lustig et al. [14] clearly confirmed the efficiency of the compressed sensing algorithm, which has led to many other CS approaches using various sparsifying transform, and optimization algorithms, etc. For example, to deal with several hyperparameters to trade off between sparsity and data fidelity terms. Many research efforts have been made to quantity such trade- off, provide different forms of reconstruction, even to propose hyperparameter free reconstruction method [63–70].

## Advanced formulation for compressed sensing MRI

### Non-Cartesian compressed sensing MRI

Compressed sensing with non Cartesian sampling has been also extensively stud- ied, since they are really a great combination given the sampling behavior of non Cartesian sampling schemes and the incoherence requirement in compressed sens- ing reconstruction. Aside from the radial and spiral sampling patterns discussed before, the works in [71–83] have fo- cused on designing better sampling trajectories with good incoherent properties. For example, Haldar et al. [82] and Puy et al. [83] proposed random phase encod- ing scheme to maximize the incoherency of the sensing matrix. However, one of the main technical issues associated with non-cartesian compressed sensing MRI is that the fast reconstruction trick shown in (21) cannot be used, which increases the overall computational time.

### Combination of parallel imaging with CS

Recall that parallel MRI (pMRI) [1, 3] exploits the diversity in the receiver coil sen- sitivity maps that are multiplied by an unknown image. This provides additional spatial information for the unknown image, resulting in accelerated MR data acquisition through k-space sample reduction. Because the aim of the parallel imaging and CS approaches is similar, extensive research efforts have been made to syner- gistically combine the two for further acceleration [18, 20, 38, 40, 84].

One of the most simplest approaches can be a SENSE type approach that explic- itly utilizes the estimated coil maps to obtain an augmented compressed sensing problem [18, 20, 38, 84]. More specifically, if the coil sensitivity is known and given by the sensitivity[*S*^*i*^], i = 1, ⋯, *C*,then the SENSE type compressed sensing MRI problem can be formulated as

22 $$ \underset{x}{\min}\frac{1}{2}{\sum}_{i=1}^C{\left\Vert {b}^i- DF\left[{S}^i\right]x\right\Vert}^2+\uplambda {\left\Vert \Psi \mathrm{x}\right\Vert}_1 $$

The optimization framework is the standard optimization framework under sparsity constraint, so proximal optimization algorithms can be used to solve this problem.

On the other hand, l_1_-SPIRiT (l_1_-iTerative Self-consistent Parallel Imaging Re- construction) [40] utilizes the GRAPPA type constraint as an additional constraint for a compressed sensing problem:

23 $$ {\displaystyle \begin{array}{c}\underset{x}{\min}\kern.5em {\left\Vert \Psi \mathrm{x}\right\Vert}_1\\ {}\kern.5em \mathrm{subject}\ \mathrm{to}\kern.5em {\left\Vert {b}^i- DF\left[{S}^i\right]x\right\Vert}^2<\epsilon, \kern1em i=1,\dots, C\\ {}\mathrm{x}=\mathrm{Mx}\end{array}} $$

where *M* is an image domain GRAPPA operator. In both approaches, an accurate estimation of coil sensitivity maps or GRAPPA kernel is essential to fully exploit the coil sensitivity diversity. To address this problem, Uecker et al. [85] developed a novel eigen space method to extract the coil sensitivity maps directly from the k-space data, which is one of the most popular methods widely used by MR researchers.

### Blind compressed sensing MR using dictionary learning

Blind compressed sensing approaches attempted to simultaneously reconstruct the underlying image as well as the sparsifying transform from highly undersampled measurements. Ravishankar and his colleagues pioneered two distinct approaches - synthesis dictionary learning [48] and analysis transform learning [86] - when the underlying sparsifying transform is unknown a priori.

#### Synthesis dictionary-based BCS

More specifically, let *P*_*j*_, j = 1 …, N represents the operator that extracts a m- dimensional patch as a vector P_*j*_x ∈ C^m^ from the image x, where N denotes the number of patches. Then, dictionary learning is to find the unknown dictionary $D\in {\mathrm{R}}^{m\times \mathrm{Q}}$ and the corresponding sparse coefficient matrix $C\in {\mathrm{C}}^{m\times \mathrm{n}}$ such that Y = DC, where *Y* = [*P*_1_*x P*_2_*x*…, *P*_*N*_*x*]

The synthesis model allows each patch *P*_*j*_x to be approximated by a linear combina- tion *D*c_*j*_ of a small number of columns from a dictionary $D\in {\mathrm{C}}^{n\times \mathrm{K}}$, where c_*j*_ ∈ C^K^ is sparse. The columns of the learnt dictionary (represented by d_*k*_, 1 ≤ k ≤ K) in (P0) are additionally constrained to be of unit norm in order to avoid the scaling ambiguity. The dictionary, and the image patch, are assumed to be much smaller than the image. This model can be used as a signal model, and Ravishankar et al. [48] proposed the following patch-based dictionary learning regularizer:

24 $$ \mathrm{R}\left(\mathrm{x}\right)=\underset{D,C}{\min }{\sum}_{j=1}^N{\left\Vert {P}_jx-{D}_{C_j}\right\Vert}^2,s.t,\left\Vert {d}_i\right\Vert =1,\forall \mathrm{i},{\left\Vert {C}_j\right\Vert}_0\le k\forall \mathrm{j} $$

where *C*_*j*_ and *d*_*j*_ denotes the j-th column of *C* and *D*, respectively. Then, the associated BCS formulation is given by

25 $$ (P0):\underset{x,D,C}{\min }v{\left\Vert Ax-b\right\Vert}_2^2+\sum \limits_{j=1}^N{\left\Vert {P}_jx-{D}_{C_j}\right\Vert}^2,s.t,\left\Vert {d}_i\right\Vert =1\forall \mathrm{i},{\left\Vert {C}_j\right\Vert}_0\le s\forall \mathrm{j} $$

where *A*: *=* *DF* denotes the downsampled Fourier transform.

To address the optimization problem (P0), Ravishankar et al. [48] employed the following two-step alternating minimization algorithm. First, the following mini- mization problem is solved by fixing x:

26 $$ \underset{D,C}{\min}\sum \limits_{j=1}^N{\left\Vert {P}_jx-{D}_{C_j}\right\Vert}^2,s.t,\left\Vert {d}_i\right\Vert =1\forall \mathrm{i},{\left\Vert {C}_j\right\Vert}_0\le K\forall \mathrm{j} $$

The K-SVD algorithm [87] was used to learn the dictionary. For a given dictionary

*D*, the image update can be done by

27 $$ \underset{x}{\min}\left\{\ v{\left\Vert Ax-b\right\Vert}_2^2+{\sum}_{j=1}^N{\left\Vert {P}_jx-{D}_{C_j}\right\Vert}^2\right\} $$

These steps are alternated until convergence. The dictionary learning MRI have shown superior image reconstructions for MRI, as compared to non-adaptive compressed sensing schemes.

#### Sparsifying transform-based BCS

However, the BCS Problem (P0) is both non-convex and NP-hard. Approximate iterative algorithms for (P0) typically solve the synthesis sparse coding problem re- peatedly, which makes them computationally expensive. In order to overcome some of the aforementioned drawbacks of synthesis dictionary-based BCS, Ravishankar et al. [86] proposed to use the sparsifying transform model in this work. Sparsify- ing transform learning has been shown to be effective and efficient in applications, while also enjoying good convergence guarantees. Specifically, they used the follow- ing transform learning regularizer:

28 $$ \mathrm{R}\left(\mathrm{x}\right)=\underset{W,C}{\min }{\sum}_{j=1}^N{\left\Vert W{P}_jx-{C}_j\right\Vert}^2+\uplambda \mathrm{Q}\left(\mathrm{W}\right),\mathrm{s}.\mathrm{t}.{\left\Vert B\right\Vert}_0\le s $$

where W ∈ C^{m\× m} denotes the unknown transform, the function Q(W) is a regualizer for the transform given by

$$ \mathrm{Q}\left(\mathrm{W}\right)=-\log \left|\det W\right|+0.5{\left\Vert W\right\Vert}_F^2 $$

The − log |det*W*| penalty eliminates degenerate solutions such as those with repeated rows. The ‖*W*‖_*F*_penalty helps remove a scale ambiguity in the solution.

Then, with additional constraint ‖*x*‖ ≤ *E*, the overall optimization problem is given by

29 $$ (P1):\underset{x,W,C}{\min }v{\left\Vert Ax-b\right\Vert}_2^2+\sum \limits_{j=1}^N{\left\Vert W{P}_jx-{C}_j\right\Vert}^2+\uplambda \mathrm{Q}\left(\mathrm{W}\right),s.t.{\left\Vert \mathrm{C}\right\Vert}_0\le s,\left\Vert x\right\Vert \le E, $$

where *A* *=* *DF* again denotes the downsampled Fourier transform. One of the important advantages of (P1) is that there exists a closed-form update for W, so the computationally expensive dictionary learning step can be avoided.

## K-t methods for compressed sensing dynamic MRI

Dynamic MRI is a technique to acquire temporally varying MR sequences such as cardiac cine, perfusion, time-resolved angiography, functional MRI, etc. In dynamic MRI, there exists significant redundancies along the temporal directions, which can be extensively studied in various compressed sensing approaches.

### k-t SPARSE

The k-t SPARSE by Lustig et al. [88] is an earliest version of compressed sensing dynamic MRI. Recall that the k-space measurement *b(k, t)* at time t is given by

30 $$ b\left(k,t\right)=\int \gamma \left(s,t\right){e}^{-j2\pi ks} ds $$

Another application of the Fourier transform along the temporal direction

31 $$ \gamma \left(s,\mathrm{t}\right)=\int p\left(s,f\right){e}^{-j2\pi ft} df, $$

results in the following 2-D Fourier relationship:

32 $$ b\left(k,\mathrm{t}\right)=\iint p\left(s,f\right){e}^{-j2\pi \left( kl+ ft\right)} dsdf, $$

where ρ(s, f) denotes the temporal Fourier transform of γ(s, t).

Note that *p*(*s*, *f*) is usually sparse because the periodic motions from heart or slow varying motions from fMRI can be easily sparsified using the temporal Fourier transform. Accordingly, the k-t data can be represented as mapping from the spatial- temporal image:

33 $$ b= A\rho $$

where *A*: *=* *DF* and *D* is a k-t downsampling pattern, and *F* now becomes a 2D Fourier transform. In addition to the temporal Fourier transform, the authors in [88] used the wavelet transform in the spatial dimension to exploit the spatial redundancy. Then, k-t SPARSE is formulated based on the following optimization problem:

34 $$ \underset{\gamma }{\min }{\left\Vert b- DF\gamma \right\Vert}_2^2+\uplambda {\left\Vert W\gamma \right\Vert}_1 $$

where *W* denotes the spatial wavelet transform, respectively.

### K-t FOCUSS

Preliminary CS dynamic MRI approaches [36, 88] were seemingly different from the classical k-t approach such as k-t BLAST/SENSE [5]. One of the most impor- tant contributions of the k-t FOCUSS by Jung et al. [18, 20] was to reveal that the compressed sensing dynamic MRI is not very different from the classical k-t approaches, but rather it can be obtained by a very simple modification of the classical k-t BLAST/SENSE to ensure significant performance improvement.

More specifically, rather than using wavelet transform, k-t FOCUSS exploited the x-f domain sparsity. Then, a standard compressed sensing formulation would be:

35 $$ \underset{\rho }{\min }{\left\Vert b- DF\rho \right\Vert}_2^2+\uplambda {\left\Vert \rho \right\Vert}_1 $$

to enforce the sparseness in x-f image *ρ*. However, there exists two additional novel- ties in the k-t FOCUSS. First, rather than directly enforcing the sparseness of the x-f image, the k-t FOCUSS further sparsifies the x-f image using the initial estimate.

Specifically, let ρ_0_ be the predictable initial estimate of ρ. Then, the residual

36 $$ \mathrm{x}=\uprho -\uprho 0 $$

should be much more sparse than the original spatio-temporal image. Second, rather than directly using the l_1_ minimization, k-t FOCUSS employed the reweighted norm approaches. This results in the following equivalent minimization problem:

37 $$ \underset{x,v}{\min }{\left\Vert b-A{\rho}_0- Ax\right\Vert}_2^2+\uplambda \frac{1}{2}{\sum}_{i=1}^n\left[\frac{{x_i}^2}{v_i}+{v}_i\right], $$

Then, the normal equations with respect to x and v are given by

$$ -\frac{{x_i}^2}{{v_i}^2}+1=0 $$

$$ -{F}^H\left(b-A{\rho}_0- Ax\right)+\uplambda {V}^{-1}x=0 $$

where V is a diagonal matrix whose i-th diagonal element is v_*i*_. This result in the following FOCUSS iteration:

38 $$ {\uprho}^{\left(\mathrm{n}+1\right)}={\uprho}_0+{\mathrm{W}}^{\left(\mathrm{n}\right)}{\mathrm{A}}^{\mathrm{H}}{\left({\mathrm{A}}^{\mathrm{H}}{\mathrm{W}}^{\left(\mathrm{n}\right)}\mathrm{A}+\uplambda \mathrm{I}\right)}^{-1}\left(\mathrm{v}-\mathrm{A}{\uprho}_0\right) $$

where the weighting matrix is given by

$$ {W}^{\left(n+1\right)}=\operatorname{diag}\left({\left|{x}^{\left(n+1\right)}\right|}^{\frac{1}{2}}\right). $$

One of the most powerful observations in [18, 20] was that the first iteration of (38) has very similar form to the classical k-t BLAST/SENSE algorithm, except the power factor of weighting matrix. This observation led to an innovative idea to con- vert k-t BLAST/SENSE to compressed sensing approach. More specifically, by using incoherence sampling patterns, multiple iterations and correct weighting factor for the diagonal matrix, the authors of k-t FOCUSS [18, 20] clearly demonstrated the performance improvement. This observation suggested that the improvement by the classical k-t algorithms such as k-t BLAST/SENSE [5] was not from the Bayesian perspective as the original authors of [5] had claimed, but indeed is originated from exploiting the sparsity int the spatio-temporal domain [18, 20]. Furthermore, by simply modifying the weight factor and sampling patterns, several additional iter- ation can significantly improve the performance of k-t BLAST/SENSE.

Another powerful aspect of k-t FOCUSS was that the idea can be easily extended to exploit the sparsity in other transform domains. For example, the residual step in (36) can be interpreted as sparsity promoting step by subtracting the temporal mean images. Thus, Jung et al. [20] proposes motion estimated and compensated modification of k-t FOCUSS to make the residual signal much sparser. More specifi- cally, rather than subtracting the temporal mean values, they subtracted the motion estimated frame. Note that motion estimation and compensation (ME/MC) is an essential step in video coding that uses motion vectors to exploit the temporal re- dundancies between frames [89, 90]. In order to employ ME/MC within dynamic MRI, there are several technical issues to address. First, at least one reference frame is required. This issue can be easily resolved if we acquire fully sampled data in one frame as often done in dynamic MRI. The main technical difficulty, however, comes from the existence of the low quality current frame. Fortunately, this issue can be addressed using an additional reconstruction step before the ME/MC [20].

In addition, the spatio-temporal signals can be further sparsified using data-driven transform:

$$ \uprho =\mathrm{Dc}, $$

where D denotes the learned temporal dictionary based from the images, whereas c denotes the coefficients. Then, the imaging problem can be formulated as

39 $$ \underset{c,v,D}{\min }{\left\Vert b-A{\rho}_0-A{D}_C\right\Vert}_2^2+\uplambda \frac{1}{2}{\sum}_{i=1}^n\left[\frac{{C_i}^2}{v_i}+{v}_i\right], $$

For example, in order to find the temporal basis that can sparsify ρ, Jung et al. [18, 20] performed the the singular value decomposition (SVD) after the image reconstruction, then the new dictionary is used to estimate the new coefficients. This procedure is closely related to the partial separable function (PSF) [91] and k-t SLR (k-t sparse and low-rank decomposition) [49], which will be reviewed soon.

### Partially separable function (PSF) approach

In the PSF model by Liang et al. [91], the k-t samples are assumed to be decomposed in the following form

$$ b\left(k,t\right)=\sum \limits_{l=1}^L{\Psi}_l(k){\varnothing}_l(t) $$

for some data dependent spectral and temporal basis function ${\left\{{\Psi}_l(k)\right\}}_{l=1}^L$ and ${\left\{{\varnothing}_l(t)\right\}}_{l=1}^L$ . Thanks to the partially separable assumption, the so-called Casorati Matrix B for the fully sampled k-t data given by

$$ \mathrm{B}=\left[\begin{array}{ccc}b\left({k}_1,{t}_1\right)& \cdots & b\left({k}_1,{t}_n\right)\\ {}\vdots & \ddots & \vdots \\ {}b\left({k}_m,{t}_1\right)& \cdots & b\left({k}_m,{t}_n\right)\end{array}\right] $$

has at most rank L.

In dynamic CS MRI, many of the k-t samples are missing and we are interested in finding the missing components. Hence, by utilizing the low-rankness of the Ca-sorati matrix, the missing k-t samples can be estimated using a low rank matrix completion algorithm. In particular, the authors in [92, 93] proposed the following matrix factorization approach:

40 $$ \left\{\widehat{U},\widehat{V}\right\}=\mathit{\arg}\underset{U,V}{\min }{\left\Vert A\left(U{V}^H\right)-B\right\Vert}^2 $$

where *A* denotes the k-t sampling operator that indicates the missing samples by 0, and $U\in {\mathrm{C}}^{m\times \mathrm{L}}$, $V\in {\mathrm{C}}^{m\times \mathrm{L}}$ is used for the low rank matrix factorization *B* = *U V* ^*H*^, and the optimization is performed for U and V alternatingly by fixing the other matrix using the previous estimate. Because the exact rank of B is not known, the authors proposed the incremented powerFactorization (IRFP) algorithm [92], where (40) starts with *L* = 1 by increasing order with the initialization of the power factorization of *L* + 1 from that of *L*. To avoid an overfitting, the algorithm is terminated as soon as the data fidelity is below some threshold values.

### K-t SLR: K-t sparse and low rank approach

k-t Sparse and Low Rank Approach (k-t SLR) model by Lingala et al. [49] is a more systematic way of learning both basis and sparse coefficients. In this approach, the spatio-temporal signal ρ(x, t) is first rearranged in a matrix form

41 $$ \uptau =\left[\begin{array}{ccc}\rho \left({x}_1,{t}_1\right)& \cdots & \rho \left({x}_1,{t}_n\right)\\ {}\vdots & \ddots & \vdots \\ {}\rho \left({x}_m,{t}_1\right)& \cdots & \rho \left({x}_m,{t}_n\right)\end{array}\right] $$

Then, using the low rank prior, the optimisation problem can be formulated as

$$ \underset{\Gamma}{\min }{\left\Vert b-A\left(\Gamma \right)\right\Vert}^2+\uplambda \upvarphi \left(\Gamma \right) $$,

where *A*: *=* *DF* is now a downsampled Fourier transform. Here, the rank prior is approximated using the general class of Schatten p-functionals, specified by

$$ \upvarphi \left(\Gamma \right)={\left\Vert \Gamma \right\Vert}_p^p=\sum \limits_i{\upsigma}_i^p, $$

where {σ_*i*_} denotes the singular values of Γ.

In dynamic imaging applications, the images in the time series may have sparse wavelet coefficients or sparse gradients. In addition, if the intensity profiles of the voxels are periodic (e.g., cardiac cine), they may be sparse in the Fourier domain. Based on this observation, Lingala et al. [49] proposed additional sparsity inducing penalty in specified basis sets along with the low-rank property to further improve the recovery rate. Specifically, they chose the 2-D wavelet transform to sparsify each of the images in the time series, while can be a 1-D Fourier transform to exploit the pseudo-periodic nature of motion. Then, the resulting composite minimization problem can be formulated as

42 $$ \underset{\Gamma}{\min }{\left\Vert b-\mathcal{A}\left(\Gamma \right)\right\Vert}^2+{\lambda}_1{\left\Vert \Gamma \right\Vert}_p^p+{\lambda}_2{\left\Vert \sqrt{\sum \limits_{i=0}^{q-1}{\left|{\Phi}_i^H\Gamma {\Psi}_{\mathrm{i}}\right|}^2}\right\Vert}_1 $$

## K-space structured low-rank approaches

### Basic theory

Compared to the standard compressed sensing approaches, k-space structured low- rank approaches such as SAKE [55], LORAKS [56], ALOHA [57, 59, 60] and GIRAF [61] are relatively new, but has significant potentials in MRI imaging. These ap- proaches are all derived by the k-space convolution relationship and can be used for both static and dynamic imaging. So we first discuss a matrix representation of the convolution. For simplicity, we will consider the 1-D notation.

Specifically, consider a fully sampled k-space measurement from the multi-channel coils:

$$ {y}^i=F{\gamma}^i,i=1,\cdots, C $$

where γ^*i*^ denotes the unknown i-th coil images and y^*i*^ corresponds to its k-space data. Note that the matrix representation of a k-space convolution of y^*i*^ with a d-tap filter h is given by

43 $$ {z}^i=\mathcal{C}\left({y}^i\right)\overline{h} $$

where $\mathcal{C}\left({y}^i\right)$ denotes the convolution matrix constructed by the vector y^*i*^:

44 $$ \mathcal{C}\left({y}^i\right)=\left[\begin{array}{cccc}\vdots & \vdots & \ddots & \vdots \\ {}{y}^i\left[-1\right]& {y}^i\left[0\right]& \cdots & {y}^i\left[d-2\right]\\ {}{y}^i\left[0\right]& {y}^i\left[1\right]& \cdots & {y}^i\left[d-1\right]\\ {}{y}^i\left[1\right]& {y}^i\left[2\right]& \cdots & {y}^i\left[d\right]\\ {}\vdots & \vdots & \ddots & \vdots \\ {}{y}^i\left[n-d\right]& {y}^i\left[n-d+1\right]& \cdots & {y}^i\left[n-1\right]\\ {}{y}^i\left[n-d+1\right]& {y}^i\left[n-d+2\right]& \cdots & {y}^i\left[n\right]\\ {}\vdots & \vdots & \ddots & \vdots \end{array}\right] $$

and h¯ denotes a vector that reverses the order of the elements. If we extract the n − d-rows of the convolution matrix with n − d > d, we can obtain the following Hankel structured matrix:

45 $$ {\mathcal{H}}_{\mathrm{d}}\left({y}^i\right)=\left[\begin{array}{cccc}{y}^i\left[0\right]& {y}^i\left[1\right]& \cdots & {y}^i\left[d-1\right]\\ {}{y}^i\left[1\right]& {y}^i\left[2\right]& \cdots & {y}^i\left[d\right]\\ {}\vdots & \vdots & \ddots & \vdots \\ {}{y}^i\left[n-d\right]& {y}^i\left[n-d+1\right]& \cdots & {y}^i\left[n-1\right]\end{array}\right] $$

By defining Y = [y^1^, ⋯, *y*^*C*^], we can further defined the extended Hankel matrix

46 $$ {\mathcal{H}}_{d\mid C}(Y){h}_{\left(i,k\right)}=\left[{\mathcal{H}}_d\left({y}^1\right)\cdots {\mathcal{H}}_d\left({y}^C\right)\right] $$

In the following, we will explain how these Hankel structured matrix has been utilised for accelerated MRI.

### SAKE

A calibrationless parallel imaging reconstruction method, termed simultaneous au- tocalibrating and k-space estimation (SAKE), is a data-driven, coil-by-coil recon- struction method that does not require a separate calibration step for estimating coil sensitivity information [55]. SAKE is based on the following observation in GRAPPA:

47 $$ {\sum}_{k\ne i}^C{y}^k\ast {w}_{\left(k,i\right)}={y}^i $$

which implies that the i-th k-space measurement can be represented as the linear combination of the filtered k-space data from other coils. In matrix form, this recursive relationship implies the existence of the null space of the Hankel matrix.

48 $$ {\mathcal{H}}_{d\mid C}(Y){h}_{\left(i,k\right)}=\left[{\mathcal{H}}_d\left({y}^1\right)\cdots {\mathcal{H}}_d\left({y}^C\right)\right]{\mathrm{h}}_{\left(\mathrm{i},\mathrm{k}\right)}=0 $$

In other word, *H*_*d*|*C*_(Y) is low-ranked. Therefore, SAKE solves the following low- rank matrix completion problem to interpolate the missing k-space data:

$$ \underset{M}{\min}\kern0.50em \operatorname{rank}\left(\mathcal{H}(M)\right) $$

$$ \mathrm{subject}\ \mathrm{to}\kern0.50em {\mathrm{P}}_{\Omega}\left({m}^i\right)={P}_{\Omega}\left({y}^i\right),i=1,\cdots, C, $$

where P_Ω_(·) denotes the projection on the measured k-space samples on the index set Ω. The problem was solved using the iterative singular value shrinkage method [94].

### LORAKS

The low-rank modeling of local-space neighborhoods (LORAKS) [56] was inspired by the finite support condition. More specifically, if the object γ has finite support, we can easily find the function w such that

$$ \upgamma \mathrm{w}=0, $$

This results in a convolution relationship in k-space

$$ \mathrm{y}\ast \mathrm{h}=0\Rightarrow \mathcal{H}\left(\mathrm{y}\right)\mathrm{h}=0, $$

where y and h denote the Fourier spectrum of γ and w, respectively. Therefore, this gives a single channel version of the low-rank condition, which results in the following rank minimization problem:

$$ \underset{m}{\min}\kern0.5em \operatorname{rank}\left(\mathcal{H}(m)\right) $$

$$ \mathrm{subject}\ \mathrm{to}\ {\mathrm{P}}_{\Omega}(m)={P}_{\Omega}\left(\widehat{y}\right), $$

### ALOHA and GIRAF

Annihilating filter-based low rank Hankel matrix (ALOHA) approach [57–60, 62] and GIRAF (Generic Iterative Reweighted Annihilating Filter) [61] can be considered as the full generalization of SAKE and LORAKS for general class of signals with the finite rate of innovations (FRI) for MR measurements. Moreover, the approaches have unified the parallel imaging and compressed sensing as a k- space interpolation with performance guarantees [62], and can be used for artifact correction [58]. This section describes the fundamental dual relationship between transform domain sparsity and low rankness in reciprocal domain, which is the key ingredient. For better readability, we provide here a high level description by assuming 1-D signals.

The Fourier CS problem of our interest is to recover the unknown signal x(t) from the Fourier measurement:

49 $$ \widehat{x}\left(\omega \right)=\mathcal{F}\left\{x(t)\right\}=\int x(t){e}^{- i\omega t} dt. $$

In classical Nyquist sampling, to avoid aliasing artefacts, the grid size should be at most:

$$ \Delta  =2\uppi /\uptau $$

when the support of the time domain signal x(t) is τ. Then, discrete Fourier data at the Nyquist rate is defined by:

50 $$ {\left.\widehat{x}\left[k\right]=\widehat{x}\left(\omega \right)\right|}_{\omega =\frac{2\pi k}{\tau }}. $$

We also define a length (r + 1)-annihilating filter hˆ[k] for xˆ[k] that satisfies

51 $$ \left(\widehat{h}\ast \widehat{x}\right)\left[k\right]={\sum}_{p=0}^r\widehat{h}\left[p\right]\widehat{x}\left[k-p\right]=0,\kern0.5em \forall k. $$

The existence of the minimum length finite length annihilating filter has been ex- tensively studied for FRI signals [95–97]. Let r + 1 denotes the minimum size of annihilating filters that annihilates discrete Fourier data $\widehat{\mathrm{x}}\left[\mathrm{k}\right]$. Then, a d-tap anni- hilating filter h with d > r + 1 can be easily obtained by convolving an appropriate size FIR filter with the minimum length annihilating filter. In matrix form, this is equivalent to

52 $$ {\mathcal{H}}_{\mathrm{d}}\left(\widehat{x}\right)\overline{h}=0 $$

where the Hankel structure matrix ${\mathcal{H}}_{\mathrm{d}}\left(\hat{x}\right)$ is constructed as

53 $$ {\mathcal{H}}_{\mathrm{d}}\left(\widehat{x}\right)=\left[\begin{array}{cccc}\widehat{x}\left[0\right]& \widehat{x}\left[1\right]& \cdots & \widehat{x}\left[d-1\right]\\ {}\widehat{x}\left[1\right]& \widehat{x}\left[2\right]& \cdots & \widehat{x}\left[d\right]\\ {}\vdots & \vdots & \ddots & \vdots \\ {}\widehat{x}\left[n-d\right]& \widehat{x}\left[n-d+1\right]& \cdots & \widehat{x}\left[n-1\right]\end{array}\right] $$

Assume that min(n − d + 1, d) > r. Then, we can show the following low rank property [62]:

54 $$ \mathrm{RANK}\mathcal{H}\left(\widehat{x}\right)=r, $$

Thanks to the low-rankness of the associated Hankel matrix, the missing k-space data can be easily interpolated using the following low-rank matrix completion [62]:

55 $$ {\displaystyle \begin{array}{c}\underset{m\in {\mathbb{C}}^n}{\operatorname{minimize}}\ \mathrm{RANK}\ \mathcal{H}(m)\\ {}\mathrm{subject}\ \mathrm{to}\ {\mathrm{P}}_{\Omega}(m)={P}_{\Omega}\left(\widehat{x}\right),\end{array}} $$

where P_Ω_ is the projection operator on the sampling location Ω. Moreover, as shown in [62], the low-rank matrix completion approach (55) does not compromise any optimality compared to the standard Fourier CS.

Note that signals may not be sparse in the image domain, but can be sparsified in a transform domain. In fact, this was the main idea of the compressed sensing. Specifically, the signal x of our interest is a non-uniform spline that can be represented by:

56 $$ \mathrm{Lx}=\mathrm{w} $$

where L denotes a constant coefficient linear differential equation that is often called the continuous domain whitening operator in [98, 99]:

57 $$ \mathrm{L}:= {\mathrm{a}}_K{\partial}^K+{\mathrm{a}}_{K-1}{\partial}^{K-1}+\dots +{\mathrm{a}}_1\partial +{\mathrm{a}}_0 $$

and w is a continuous sparse innovation:

58 $$ w\left(\mathrm{t}\right)={\sum}_{j=0}^{r-1}{c}_j\delta \left(t-{t}_j\right). $$

For example, if the underlying signal is piecewise constant, we can set L as the first differentiation. In this case, x corresponds to the total variation signal model. Then,

by taking the Fourier transform of (56), we have

59 $$ \widehat{z}\left(\omega \right):= \widehat{l}\left(\omega \right)\widehat{x}\left(\omega \right)={\sum}_{j=0}^{r-1}{a}_j{\mathrm{e}}^{-\mathrm{i}\upomega {\mathrm{x}}_{\mathrm{j}}} $$

where

60 $$ \widehat{l}\left(\omega \right)={a}_K{\left( i\omega \right)}^K+{a}_{K-1}{\left( i\omega \right)}^{K-1}+\cdots +{a}_1\left( i\omega \right)+{a}_0 $$

Accordingly, the Hankel matrix $\mathcal{H}\left(\hat{z}\right)$ from the weighted spectrum zˆ(ω) satisfies the following rank condition:

$$ \mathrm{RANK}\mathcal{H}\left(\widehat{z}\right)=r. $$

Thanks to the low-rankness, the missing Fourier data can be interpolated using the following matrix completion problem:

61 $$ {\displaystyle \begin{array}{c}\left({P}_w\right){\min}_{m\in {\mathbb{C}}^n}{\left\Vert \mathcal{H}(m)\right\Vert}_{\ast}\\ {}\mathrm{subjectto}{P}_{\Omega}(m)={P}_{\Omega}\left(\hat{l}\odot \hat{y}\right),\end{array}} $$

or, for noisy Fourier measurements $\widehat{\mathrm{y}}$,

62 $$ {\displaystyle \begin{array}{c}\left({P}_{\mathrm{w}}^{\prime}\right){\min}_{m\in {\mathbb{C}}^n}{\left\Vert \mathcal{H}(m)\right\Vert}_{\ast}\\ {}\mathrm{subjectto}\left\Vert {P}_{\Omega}(m)-{P}_{\Omega}\left(\hat{l}\odot \hat{y}\right)\right\Vert \le \delta \end{array}} $$

where ⊙ denotes the Hadamard product, $\widehat{\mathrm{l}}$ and $\widehat{\mathrm{x}}$ denotes the vectors composed of full samples of $\widehat{\mathrm{l}}$(ω) and $\widehat{\mathrm{x}}$(ω), respectively. After solving (P_*w*_), the missing spec- tral data xˆ(ω) can be obtained by dividing by the weight, i.e. $\widehat{x}\left(\omega \right)=\mathrm{m}\left(\upomega \right)/\widehat{l}\left(\omega \right)$ assuming that $\widehat{l}\left(\omega \right)\ne 0$.

**Fig. 3:**
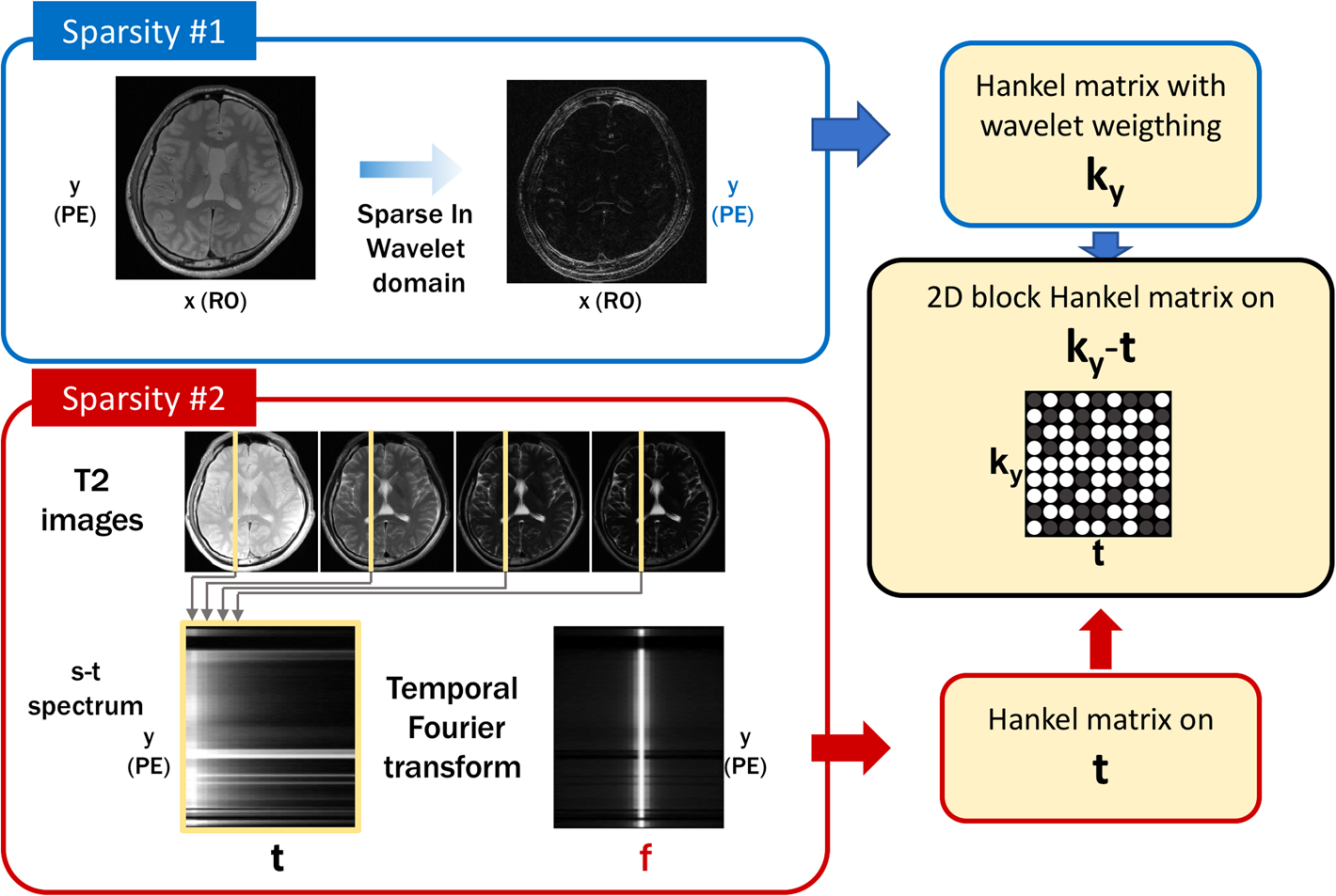
Construction of Hankel matrix for MR parameter mapping [59]

The idea can be generalized for any transform domain sparse signals as long as the transform can be represented using shift-invariant filters. Wavelet domain sparse signal belongs to this class. In this case, the weight kernel in the Fourier domain is obtained as the spectrum of the subband filters [57–60, 62]. For example, Fig. 3 showed the construction of the Hankel matrix for the MR parameter mapping, where the k-space weighting from wavelet weighting is applied only along the phase encoding direction, whereas no weighing is applied along the t-domain since the correspond temporal spectrum is already sparse [59].

The idea can be also easily generalised to the parallel imaging by exploiting (19). Specifically, (19) can be equivalently represented using the matrix representation:

63 $$ {\mathcal{H}}_{\mathrm{d}}\left({\widehat{\gamma}}^i\right){\widehat{s}}^j={\mathcal{H}}_d\left({\widehat{\gamma}}^j\right){\widehat{s}}^i,\forall \mathrm{i}\ne \mathrm{j}, $$

This implies that an exten^ded Hankel matrix ${\mathcal{H}}_{\mathrm{d}\mid \mathrm{C}}\left(\left[{\widehat{\gamma}}^1\cdots {\widehat{\gamma}}^C\right]\right)$ in (46) is low ranked.

**Fig. 4:**
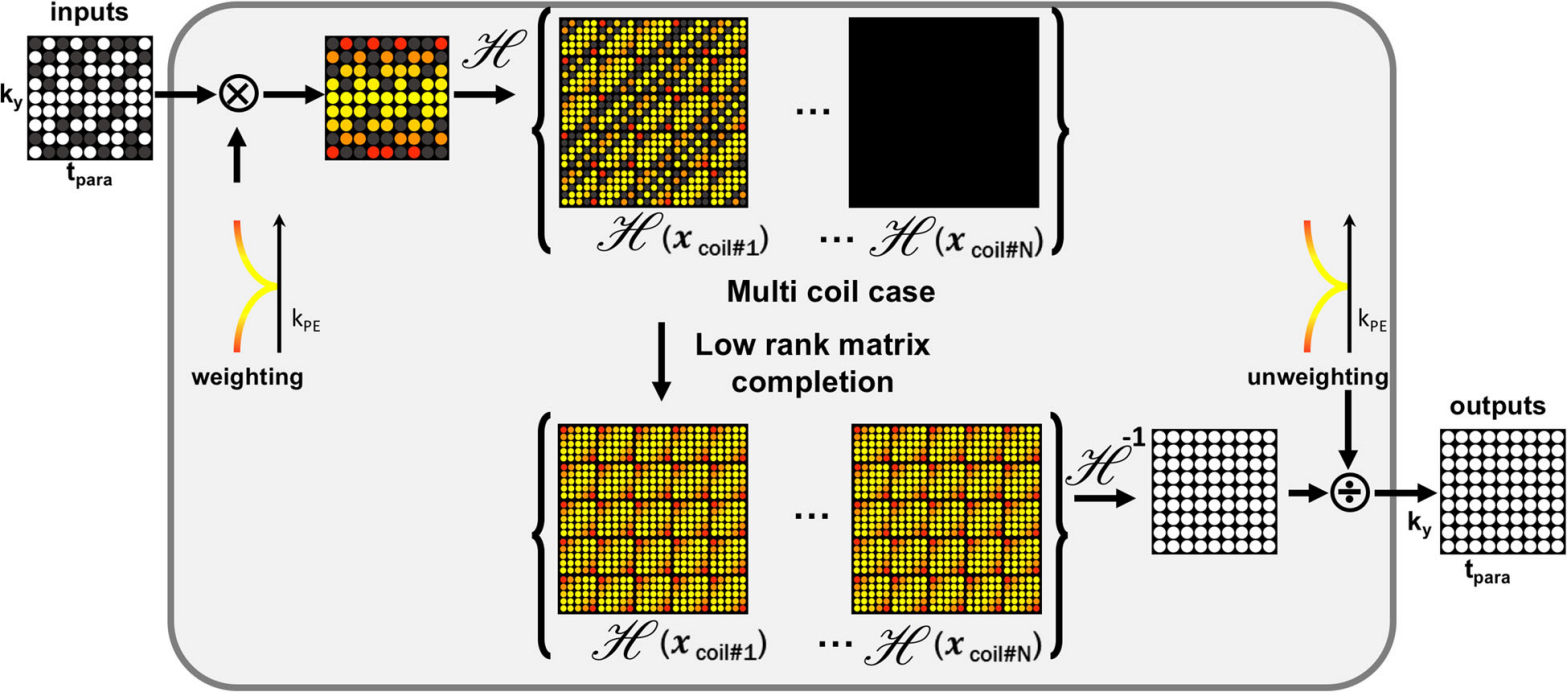
Construction of Hankel matrix for multi-channel filter data for the case of MR parameter mapping [59]

For example, the multi-channel construction of Hankel matrix for MR parameter mapping [59] is shown in Fig. 4.

One of the most important advantages of the Hankel matrix formulation is that the coil diversity can be readily exploited in addition to the image domain redundancy in a unified framework. This makes the separate coil sensitivity estimation unnecessary.

## Clinical applications

Since the compressed sensing MRI allows significant acceleration of MR acquisition, it has been extensively applied for various clinical applications such as fast cardiac MRI, whole heart MRI, dynamic contrast enhanced (DCE)-MRI, diffusion MRI, spectroscopic, etc., that usually require significant acquisition time using standard methods.

For example, Otazo et al. [84] applied the CS method to the first pass cardiac perfusion MRI and demonstrated feasibility of 8-fold acceleration in vivo imaging using standard coil arrays. They showed that CS method results in similar temporal fidelity and image quality to GRAPPA with 2-fold acceleration [84]. Hsiao et al. [100] applied combined parallel imaging and compressed sensing to achieve 4D phase contrast for the quantification of cardiac flow and ventricular volumes pediatric patients during congenital heart MRI examinations. Vincent et al. [101] employed CS to evaluate LV function and volumes and found that CS strategy with the single breath hold provided similar results to multi breathhold imaging protocols.

For free-breathing contrast-enhanced multiphase liver MRI, Chandarana et al. [102] showed that a combination of compressed sensing, parallel imaging, and radial k-space sampling demonstrated the feasibility of breath-hold cartesian T1 weighted imaging. Espagnet et al. [103] employed golden-angle radial sparse parallel technique for DCE-MRI to evaluate the permeability characteristics of the pituitary gland.

For diffusion MRI, Landman et al. [104] showed that CS reconstruction using standard data can resolve crossing fibers similar to a standard q-ball approach using much richer data with longer acquisition time. Kuhnt et al. [105] showed that High Angular Resolution Diffusion Imaging (HARDI) + CS is a promising approach for fiber tractography in clinical practice.

Finally, for the spectroscopic imaging, Larson et al. [106] developed a CS method for acquiring hyperpolarized 13C data using multiband excitation pulses and achieved 2 s temporal resolution with full volumetric coverage of a mouse. Geethanath et al. [107] demonstrated a potential reduction in acquisition time by up to 80% or more for hydrogen 1 MR spectroscopic imaging using CS, with negligible loss of clinical information.

With the commercially available CS reconstruction methods, we expect to see more clinical applications of CS in the near future.

## Conclusions

Nowdays, compressed sensing has become an mature technology, as reflected by recent approval by FDA. Major vendors have started to sell the compressed sensing reconstruction softwares, and many clinical researchers have been evaluating its clinical usefulness.

Despite of this maturity, some of the main technical issues of the compressed sensing are 1) the computational complexity of the algorithm is relative high, and at high acceleration, image quality degradation is still reported. Although the recent state-of-the art CS techniques such as structured Hankel matrix approach can address the quality degradation problems, it also increases the computational complexity, which may interfere the clinical workflow.

Fortunately, for the last two years, the MR image reconstruction field have been rapidly changed thanks to the successful demonstration of of the deep learning- based MR reconstruction technologies [108–113]. The sudden popularity of deep learning approaches can be attributed to the real-time reconstruction in spite of the significant improvement of the image quality. Thus, when originally presented, these techniques were regarded as totally different technology that is nothing to do with the compressed sensing. However, recent theoretical analysis [114] showed that the deep convolutional neural network is closely related to the Hankel matrix decomposition. Therefore, we can still argue that the compressed sensing MRI has renewed interests in the form of deep learning, and it will be interesting to see how this exciting and rapidly evolving field will develop for the coming decades.

## Abbreviations

ALOHA: Annihliating filter-based low-rank Hankel matrix approach
CS: Compressed sensing
FOCUSS: FOCal Underdetermined System Solver
GRAPPA: Generalized Autocalibrating Partially Parallel Acquisitions
*l*1-SPiRIT: *l*1-iTerative Self-consistent Parallel Imaging Reconstruction
LORAKS: Low-rank modeling of local-space neighborhoods
MRI: Magnetic resonance imaging
SAKE: Simultaneous autocalibrating and k-space estimation
SENSE: Sensitivity encoding
